# Temporal and individual variation in the feeding habits of Asiatic black bears (*Ursus thibetanus*)

**DOI:** 10.1002/ece3.11562

**Published:** 2024-07-09

**Authors:** Tomoki Mori, Saki N. Mori, Shigeyuki Izumiyama

**Affiliations:** ^1^ Institute for Mountain Science Shinshu University Minami‐minowa, Kami‐Ina Japan; ^2^ Graduate School of Science and Technology Shinshu University Minami‐minowa, Kami‐Ina Japan; ^3^ Present address: Research Center for Wildlife Management Gifu University Gifu Japan

**Keywords:** diets, individual characteristics, scat, seasonal shifts, *Ursus thibetanus*

## Abstract

Foraging plays a vital role in the survival of wildlife, and shifts in food availability can impact species fitness and survival. Ursids are known to consume a wide variety of foods and are known to be opportunistic omnivores. Consequently, seasonal shifts in diet, which correspond to temporal and spatial shifts in the availability of food resources, have long captivated researchers studying the foraging behavior of Ursidae. Nevertheless, comprehensive dietary studies encompassing both the population and individual levels remain scarce. In this study, we investigated the dietary patterns of Asiatic black bears (*Ursus thibetanus*) at both the population and individual levels, using data collected through GPS collars and field surveys of individual bear scat samples in Nagano Prefecture, Japan, from 2016 to 2020. From early April to late June, bears mainly foraged on green vegetation. During this period, male and large‐bodied female bears showed a strong preference for green vegetation. Small‐bodied female bears also ate mostly green vegetation but tended to consume more fruit than other bears towards the end of this period. From June to October, bears' diets included a substantial amount of fruit, with notable peaks in fruit consumption in late June and early September. During the summer months, female bears often incorporated social insects into their diet compared to the population‐level trend. In mid‐September, the consumption of seeds from the Fagaceae family surged, becoming the primary dietary component during this period. This trend was consistently observed across the population. These findings underscore the importance conducting in‐depth dietary analyses that take into account individual characteristics such as sex, age, and body weight.

## Graphical abstract

Focusing on individual differences in the dietary patterns of Asiatic black bears in Nagano Prefecture, Japan, male and large‐bodied female bears preferred green vegetation in early summer, while small‐bodied female bears favored fruit. Additionally, female bears were observed to incorporate ants into their diet during the summer months. These findings highlight the importance of considering individual characteristics in dietary analyses.

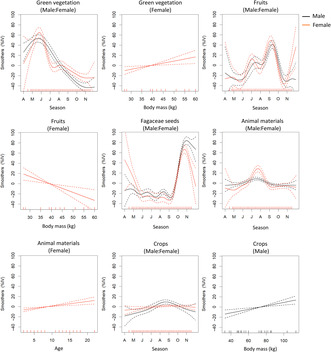

## 1 INTRODUCTION

Foraging is an essential activity for wildlife to sustain life, and understanding foraging behavior and dietary patterns help gain a better understanding of the fundamental ecology of various wild species. This knowledge is valuable for wildlife conservation and management (Putman, 1984). In the case of Ursids, seasonal diets have long been a focal point in foraging ecology due to climatic conditions significantly influencing food distribution and availability, which turn causes temporal and spatial shift in their foraging behavior (e.g., Hashimoto, 2002; Munro et al., 2006; Murphy et al., 2017; Stenset et al., 2016). Previous studies have demonstrated that most bear species exhibit flexibility in dietary preferences and are opportunistic omnivores. They are known to consume a diverse range of foods, including green vegetation, fruits, insects, and mammals, in response to changes in the plant phenology within their habitats (Baldwin & Bender, 2009; Ciucci et al., 2014; Huygens et al., 2003; Joshi et al., 1997; Koike, 2010; Munro et al., 2006; Torgersen et al., 2001). For example, Apennine brown bears (*Ursus arctos arctos*) in Italy have been observed consuming herbaceous vegetation, ants, and ungulates during spring and early summer, whereas their diet shifts to berries in the summer and hard masts and other fruits in autumn (Ciucci et al., 2014). Similarly, American black bears (*Ursus americanus*) in Canada exhibit seasonal variations in their diet, with herbaceous vegetation and ants being the primary dietary components in spring, followed by an emphasis on ants in summer and berries in autumn (Lesmerises et al., 2015). In preparation for hibernation, bears engage in intense feeding during autumn to increase fat storage, a phenomenon known as hyperphagia (Nelson et al., 1983). Additionally, food resource scarcity becomes more pronounced seasonally, particularly from late spring to summer, leading bears to face a negative energy balance and sometimes resort to consuming less preferred or risker foods, including anthropogenic food sources (e.g., Furusaka et al., 2019; Lewis et al., 2015; Yamanaka et al., 2011).

However, most studies have treated the species as a collective entity, assuming that trends in seasonal dietary changes apply uniformly within a population, while paying little attention to individual dietary variations. However, recent studies have highlighted the fact that individuals within a population exhibit distinct food preferences owing to variations in their characteristics, such as age, sex, and morphology (Lesmerises et al., 2015; Murray et al., 2017; Naganuma et al., 2020; Thiemann et al., 2011). For example, large‐bodied brown bears (*Ursus arctos*) consume more meat compared to small‐bodied bears (Costello et al., 2016), and male American black bears tend to consume more meat than their female counterparts (Hatch et al., 2019). Similarly, previous studies have indicated that smaller American black bears tend to consume more insects than their larger counterparts, likely because of the physical constraints of larger bear faces, particularly when feeding on ants (Costello et al., 2016; Welch et al., 1997). These examples of dietary divergence among bears highlight the potential for seasonal shifts in individual dietary preferences. Therefore, to comprehensively understand the seasonal shifts in bear diets, it is necessary to conduct individual‐level investigations to reveal seasonal variations in food habits. By examining both individual and population‐level dietary patterns, we can gain a more nuanced understanding of foraging behavior, which is essential for understanding critical aspects of animal ecology and behavior.

Asiatic black bears (*U. thibetanus*) inhabiting Japan are omnivores that predominantly subsist on plant materials, such as green vegetation and fruits. However, depending on the season, mammal carcasses and colonial insects can also become a significant part of their diet (Hashimoto & Takatsuki, 1997). Previous studies have demonstrated substantial seasonal variations in the dietary preferences of Asiatic black bears owing to changes in plant phenology. In spring, they consume green vegetation and/or Fagaceae hard masts, whereas green vegetation, fleshy fruits, and social insects are incorporated into their diets in summer, and fleshy fruits and hard masts are consumed in autumn (e.g., Hashimoto, 2002; Koike, 2010; Mori et al., 2018). The diets of Asiatic black bears also vary significantly at the individual level depending on the season (Mori et al., 2019; Naganuma et al., 2020). This highlights the potential for discrepancies in food habits at the population level, yielding erroneous conclusions regarding individual food habits. To accurately capture the broader patterns of seasonal variation in food habits, it is essential to conduct detailed analyses of diet shifts at the individual level among bears. Such an approach acknowledges the diversity within a population and prevents the oversimplification of assuming uniform dietary changes across all individuals. However, information regarding this aspect is scarce.

In this study, we investigated the seasonal diets of Asiatic black bears at the population and individual levels. To achieve this, we revisited the feeding habits of Asiatic black bears through a reanalysis and extension of previously published data by Mori et al. (2019). Mori et al. (2019) investigated the dietary composition of 15 GPS‐collared bears and identified seasonal patterns of dietary specialization, but found little evidence of variations based on individual attributes, such as sex and body size. In this study, we expand upon the findings by incorporating data from an additional 14 bears, resulting in a total sample size of 29 individuals (16 males and 13 females). We conducted a reanalysis of the previously collected data to further investigate individual dietary differences, as well as variations in feeding habits based on sex and body size among Asiatic black bears. Our primary hypothesis was that seasonal shifts in diet would exhibit significant difference across sex, age, and body mass, such as consumption rates within each food category, namely, green vegetation, fruits, animal material, and agricultural crops. Specifically, we predicted that (1) during the early summer, when the availability and sparse distribution of fruits are limited, large‐bodied bears, especially males, would consume more green vegetation compared to females; (2) in summer months, small‐bodied females would consume more insects compared to males, as they can achieve their nutritional needs more quickly and with less food; (3) however, all bears would heavily rely on forage seeds from the Fagaceae family during the autumn season, when these seeds are most available, as these seeds are of paramount importance to Asiatic black bears as they prepare for hibernation.

## 2 MATERIALS AND METHODS

### 2.1 Study area

**FIGURE 1:**
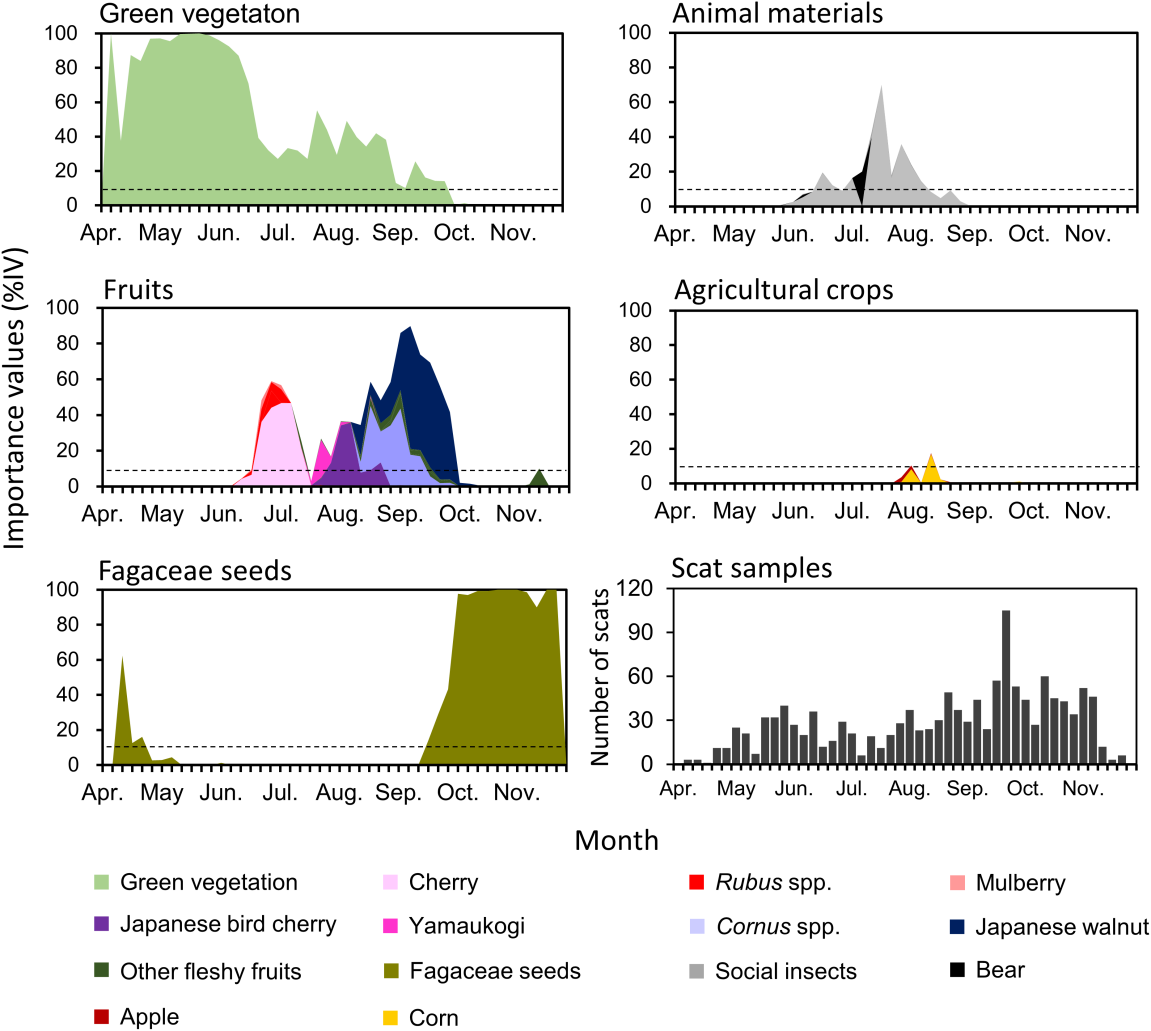
Seasonal variations in the diets of Asiatic black bears (*Ursus thibetanus*) in Nagano Prefecture, Japan, from 2016 to 2020, based on five major food categories (green vegetation, fruits, Fagaceae family, animal materials, and agricultural crops). Dotted lines demarcate the boundaries (10%IV) that signify major food sources for bears.

We conducted a field study in the northeastern part of the Central Japan Alps from 2016 to 2020, encompassing the Ina Valley to the east and the Kiso Valley to the southwest of Nagano Prefecture (35°51′ N, 137°56′ E), Japan (Figure 1). The region features steep mountainous terrain with altitudes ranging from 590 to 2956 m. The climate is temperate continental with warm summers and cold winters. During the study period, the average annual temperature was 12.7°C and the total annual precipitation was 1506 mm, as reported by the Japan Meteorological Agency (2016–2020). While the data do not separate winter snowfall from overall rainfall, the snowfall during winter is relatively rare in the study area. Much of the area, including residential and agricultural lands, lies above 700 m above sea level, with several peaks, such as Mt. Shogikashira, Mt. Utsugidake, and Mt. Gongen, exceeding 1800 m. Vegetation varies with altitude: below 700 m, streamside forests are composed of species, such as Japanese dogwood (*Cornus controversa*) and Japanese walnut (*Juglans mandshurica*). Between 800 and 1600 m, the area is characterized by plantations of larch (*Larix kaempferi*) and Japanese red pine (*Pinus densiflora*), interspersed with broad‐leaved trees, such as Mongolian oak (*Quercus crispula*), konara oak (*Q. serrata*), chestnut (*Castanea crenata*), and *Prunus* species. Above 1600 m, the subalpine zone is dominated by Russian rock birch (*Betula ermanii*) and Maries' fir (*Abies mariesii*). Bears in this region sometimes supplement their diet with crops like corn (*Zea mays*) during the summer months (Mori et al., 2019). For this study, we divided the year into four categories based on the general phenological progression of plant phenology: spring (April to May; budding and flowering of many plant species), early summer (June to July; fruiting some fleshy fruits), late summer (August to September; fruiting of a variety of fleshy fruits), and autumn (October to November; maturing of acorns).

### 2.2 GPS cluster visitation for scat sampling

We primarily obtained scat samples from bears with known sex and age. Between the spring and summer of 2015 and 2020, we fitted GPS collars (VECTRONIC Aerospace GmbH, Berlin, Germany) with a drop‐off system to 27 Asiatic black bears (14 males and 13 females) that were inadvertently captured in box traps initially set for wild boars (*Sus scrofa*) or Japanese macaques (*Macaca fuscata*), as well as in barrel traps designed for bears within the study area. Upon capture, we safely immobilized the bears using a mixture of ketamine (10 mg/kg) and xylazine (1 mg/kg). We recorded their body mass, extracted an upper premolar tooth for age estimation, and fitted them with GPS collars. All techniques and procedures employed for capturing and handling the Asiatic black bears adhered to the guidelines of the Mammal Society of Japan (2009). For two male bears captured in 2020, we estimated ages based on tooth wear and discoloration. The body mass and age of the captured bears ranged from 33 to 114 kg and 3 to 19 years for males and 27 to 60 kg and 2 to 22 years for females. Some bears were captured over multiple years, and any scats they excreted while in the traps were collected during capture. Additionally, we collected scats from two male bears that were released without collars. The GPS collars that were fitted to the bears recorded their locations every hour throughout the active bear season (excluding denning: December–May), with the exception of one collar set at 2‐h intervals because of limited battery capacity. When the tracking period extended beyond 1 year, we adjusted the fixed intervals of certain collars from 1 to 2–6 h using satellite communications to conserve battery life. At intervals of 2 weeks, we remotely downloaded location data from the active GPS collars, either directly in the field or through satellite communication.

We conducted scat sampling of individual collared bears at fixed intervals of 1–2 h between April and November (excluding the denning season) from 2016 to 2020. We plotted the location data using QGIS software (QGIS Development Team, 2016, 2018). We defined GPS clusters as locations where Asiatic black bears spent a minimum of three fixes (at least 2–4 h) (equivalent to 3–6 h) within a 20‐m radius based on the precision of GPS locations (Mori et al., 2019). We then intensively searched the cluster locations on foot within 5 days using handheld GPS devices to locate and collect individual scats. Leveraging GPS technology allowed us to pinpoint GPS cluster locations and gather evidence of animal activities, such as scats and carcasses, at either the individual or group level (e.g., Fröhlich et al., 2012; Lesmerises et al., 2015; Sand et al., 2005; Schwab et al., 2011). For a detailed description of the scat collection method, please refer to Mori et al. (2019). To ensure balanced data across individual bears, we randomly selected and surveyed two to six clusters at various times each day over a two‐week period for each bear. However, our study was limited by logical constraints, including labor limitations, collar malfunctions, and satellite errors, which occasionally hindered our ability to visit all clusters equally throughout the survey period. We carefully placed scat samples discovered at the clusters in plastic bags and frozen at −20°C until scat analysis. In cases where multiple scats were found within the same cluster, we collected all of them but only used a maximum of five randomly selected scats for analysis to prevent overrepresentation of specific food items (Dahle et al., 1998). Two bears were accompanied by cubs, prompting us to differentiate adult and cub scats based on size and exclusively collect adult scats.

In our study, we employed a GPS cluster‐based scat sampling method and did not utilize fecal DNA analysis to identify specific individuals. This implies that we may have collected scats from nontargeted, collared individuals. Of particular concern is the fact that bears tend to congregate during the mating season, which typically occurs from June to July (Naganuma et al., 2020; Yamamoto et al., 1998). During this period, it is possible that we inadvertently collected scats from partners of the collared bears. Furthermore, it is plausible that we collected scats from a different individual at the same location but during different times. To mitigate these risks, we adhered to a set of decision rules: (1) We collected scat samples only when the estimated date of scat excretion aligned with dates when the GPS cluster was formed. The freshness of the scats was assessed based on physical characteristics such as moisture content, odor intensity, and color. Fresh scats tend to be moist and have a strong odor, and a lighter color (reflecting the color of the consumed foods). These conditions are also influenced by environmental factors such as temperature, rain, and humidity. However, by considering the recent environmental conditions, we determined that only scat samples with clearly distinguishable freshness differences were judged to belong to the tracked individuals. (2) Short‐term inactivity (lasting over 7 days) in small areas during summer may be related to breeding or courtship behavior (Kozakai et al., 2013). Similarly, for bears in our study area, we observed that male and female pairs remained in a small area for over a few days during the mating season (T. Mori, personal observation). Therefore, during the mating season, we excluded GPS clusters in which bears spent over 48 h within a 50‐m radius. Taking these factors into account, we can reasonably assume that the collected scat samples originated from the targeted collared bears with a high degree of accuracy.

### 2.3 Scat analysis

We thawed each scat sample prior to analysis and carefully washed it with water while passing through two sieves (2.0 and 1.0 mm in diameter) until the water ran clear to isolate individual food items. The undigested remains obtained from the sieves were spread onto a white plastic tray featuring a 1 × 1 cm grid pattern. We estimated relative percentage volume of each food items using two methods. From 2016 to 2017, we visually assessed the percentage volume (Mealey, 1980) using a 5‐point scale: <1%, 1%–25%, 25%–50%, 50%–75%, and 75%–100%. From 2018 to 2020, we used the point‐frame method (Sato et al., 2000) to calculate occupancy, which reflects the percentage composition of food items across the surface area and saves time. We determined the proportion of each specific food item using the formula: occupancy *i* [%] = 100 × (number of intersections with food item *i*)/(total number of intersections with all food items in the scat sample). For each scat sample, we examined a minimum of 400 intersection points. These two measurement approaches have been demonstrated to be statistically compatible (Sato et al., 2000). The percentage volumes estimated using the combined methods were reassigned as 1, 12.5, 37.5, 62.5, and 87.5. Furthermore, details of the scat analysis method can be found in Mori et al. (2019).

We identified food items in bear scats to the finest possible taxonomic level. However, some species proved challenging to distinguish, and herbaceous materials, such as leaves and stems, were classified as green vegetation. The social insects and other insects were classified as insects. Additionally, we combined certain fruits, such as Japanese dogwood and large‐leaf dogwood (*C. macrophylla*) as *Cornus* spp., and Mongolian oak, Konara oak, Japanese emperor oak (*Q. dentata*), sawtooth oak (*Q. acutissima*), and Japanese chestnut (*Castanea crenata*) as the Fagaceae family. Finally, we reclassified them into five food categories: green vegetation, fruits other than the Fagaceae family (hereafter, fruits), seed of the Fagaceae family, animal materials (insects and mammals), and crops.

To investigate seasonal shifts in the bears' diets at a finer time resolution, we segmented the scat samples of each bear into 5‐day intervals for every month spanning from April 1 to November 31 (each month was divided into six sections: 1–5, 6–10, 11–15, 16–20, 21–25, and 26–; in total, 48‐time sections). This fine‐scale temporal resolution allowed us to capture detailed changes in food availability and dietary preferences, which might be missed with broader seasonal categorization. Additionally, using 5‐day intervals helped to ensure a sufficient sample size in each period, reducing the risk of over‐ or underestimation of dietary components due to sample size variability. For each section, we expressed the composition of the diets as the percentage of importance value (%IV; Mealey, 1980). This value was calculated step‐by‐step, factoring in the percentage volume, percentage frequency of occurrence, and importance value. We considered foods accounting for ≧10%IV as primary food items for bears.

### 2.4 Data analysis

The variability in bears diets across the five food categories was analyzed using generalized additive mixed models (GAMMs) with a Gaussian error distribution using the “mgcv” package in R.4.0.3 (R Development Core Team, 2020). The additive model estimates a nonparametric function, offering a flexible and effective approach for modeling both linear and nonlinear data (Wood, 2006). To streamline the analysis, the models were fitted based on sex for each food category. In the GAMMs, the response variables were %IV for each food category (green vegetation, fruits, the seed of Fagaceae family, animal materials, and crops), which were further divided by month into six sections for each bear. The fixed effects included season (48‐time sections), body mass, and age as smooth predictors. Body mass at the time of capture was used in the analysis. Since the capture period was from late May to mid‐August, it did not include the hyperphagia period when body weight increases significantly, thus, there was no significant variation in body weight. For the analysis, we assigned a numerical value ranging from 1 to 48 to each of the 48‐time sections in the analysis. Because body mass and age were highly correlated (Spearman's rank test: *r*_s_ = .75, *p* < .05) in males, we excluded age from the male model. Although the “year” should typically be included in the fixed effects, it was included as a random effect in our study to mitigate any between‐year differences due to the small sample size. To account for potential pseudo‐replication and temporal autocorrelation, we compared models that included individual ID as a random effect and models that did not, as well as models that included autocorrelation and models that did not. We conducted model selection by comparing Akaike's information criteria, which had been adjusted for the small sample sizes (AICc), and calculated Akaike weight using the MuMIn package (Barton, 2020). Models with ΔAICc <2 were considered as candidates (Burnham & Anderson, 2003). The adjusted *R* ^2^ values were extracted for each selected GAMM.

## 3 RESULTS

### 3.1 Scat sampling data

During the study period spanning from 2016 to 2020, we collected a total of 1337 scat samples from 29 individual bears (92 from two bears, 105 from 10 bears, 361 from 11 bears, 498 from 16 bears, and 281 from 15 bears). Some bears were sampled in multiple years, leading to overlapping counts in the annual datasets. The number of bears for which we successfully obtained scats for each 5‐day interval is presented in Table 1. Between 2018 and 2020, we ventured into the field to track 11, 16, and 11 collared bears, visiting 225, 260, and 132 GPS clusters, respectively. During these visits, we detected scat samples in approximately 54.6% (*n* = 123), 71.5% (*n* = 186), and 62.9% (*n* = 83) of the GPS clusters, for the respective years. In 2016 and 2017, we detected scat samples at 32 and 52 GPS clusters, respectively, for two and 10 collared bears; however, the exact number of GPS clusters from which scats could not be collected was not documented. Over 70% of the scat samples were located within 10 m of the central coordinates of the GPS clusters. We collected 24 scat samples during capture. After excluding instances where more than five scat samples originated from the same cluster, 92, 105, 356, 491, and 271 scat samples (of a total 1315 scat samples) were used for scat analysis between 2016 and 2020, respectively. The acquisition periods for the dietary data based on the collected scat samples were May 3 to October 12, May 21 to October 29, April 15 to November 13, April 24 to November 17, and April 22 to November 19 in 2016–2020, respectively.

**TABLE 1:** List of the number of collared‐Asiatic black bears (*Ursus thibetanus*) which scat collections were made in Nagano Prefecture, Japan, from 2016 to 2020.

	April						May						June						July						August						September						October						November					
	1	2	3	4	5	6	1	2	3	4	5	6	1	2	3	4	5	6	1	2	3	4	5	6	1	2	3	4	5	6	1	2	3	4	5	6	1	2	3	4	5	6	1	2	3	4	5	6
Male																																																
2016							1			1	1		1		1		1					1	1		1	1	1		1		1	1	1				1		1									
2017									1		1	1																	1	1					1		1	1			1	1						
2018		1	1	1	1			1		1	1	1		1		1		1	1			1	2	1	1	1	2	1	1	3		2	3	2	4		1	3	2	1	2		4	1				
2019					1	1	3	3		2	1			2	3	2	1	2	1		4	1	1	3	4	3		4	4	4	3	2	1	1	4	2	1			2	1		1	1	1	2		
2020					1	2	1	1		1	2	2	2		1								1	1	3		2		4	2	2	1	1	4	3	3		1	2			1			1		1	
Total	0	1	1	1	3	3	5	5	1	5	6	4	3	3	5	3	2	3	2	0	4	3	5	5	9	5	5	5	11	10	6	6	6	7	12	5	4	5	5	3	4	2	5	2	2	2	1	0
Female																																																
2016															1			1	1	1	1			1	1	1			1		1																	
2017											1				2	1	1		1		1				2			1	1				1	1	3	3	1	1	3	3		1						
2018			1		1	1	1	2	1		1	1	2		1	3		1	2	1	1	1	1		1		1	4		3		3	2	5	3	4	3	1	2	1	3	1	1	2	1			
2019									1	1	1	3	3	2	1		1	2	2		2	3	1	2	1	1		1	3	3	3	1	1	1	3	4	2	1	2	2	2	3	3	3	1			
2020					1	1		1		1	1	1	1	1			1	2	2				1		1	1	2	1	2		1	1		2	2	2	1		1									
Total	0	0	1	0	2	2	1	3	2	2	4	5	6	3	5	4	3	6	8	2	5	4	3	3	6	3	3	7	7	6	5	5	4	9	11	13	7	3	8	6	5	5	4	5	2	0	0	0

*Note*: Each number (1–6) represents one of six sections within each month from April to November (48‐time sections in total).

### 3.2 Population‐level diets

Through comprehensive scat analysis, we identified 53 distinct food items categorized into five major groups: green vegetation (7), fruits (24), seeds of the Fagaceae family (5), animal materials such as insects or mammals (12), and agricultural crops (5). At the population level, the bears primarily consumed green vegetation from early April until late June, with substantial volumes (≧10%IV) persisting until the end of September (Figure 1). Fruit consumption spanned from early June to early October, and again in mid‐November. There was a bimodal peak in fruit consumption in late June and early September. The first peak correlated with cherries (*Prunus jamasakura* and *P. leveilleana*), *Rubus* spp., and mulberry (*Morus australis*), whereas the second peak, which was larger than the first peak, correlated with Yama‐ukogi (*Eleutherococcus spinosus*), Japanese bird cherry, dogwood, Japanese walnut, and various other fruits. Seeds from the Fagaceae family came into play during early spring and autumn, with their consumption increasing dramatically from mid‐September to early October, thereafter constituting a primary food source for the bears. Insects (predominantly ants) were included from the beginning of June to late June, and again in early August. Mammals, such as Japanese serow (*Capricornis crispus*), were consumed opportunistically throughout the season. In some cases, bear cannibalism was observed during the mating season, from June to July. Corn and apple (*Malus pumila*) crops were grown between the beginning of August and the end of September.

### 3.3 Seasonal diet variations by sex, age, and individual

**FIGURE 2:**
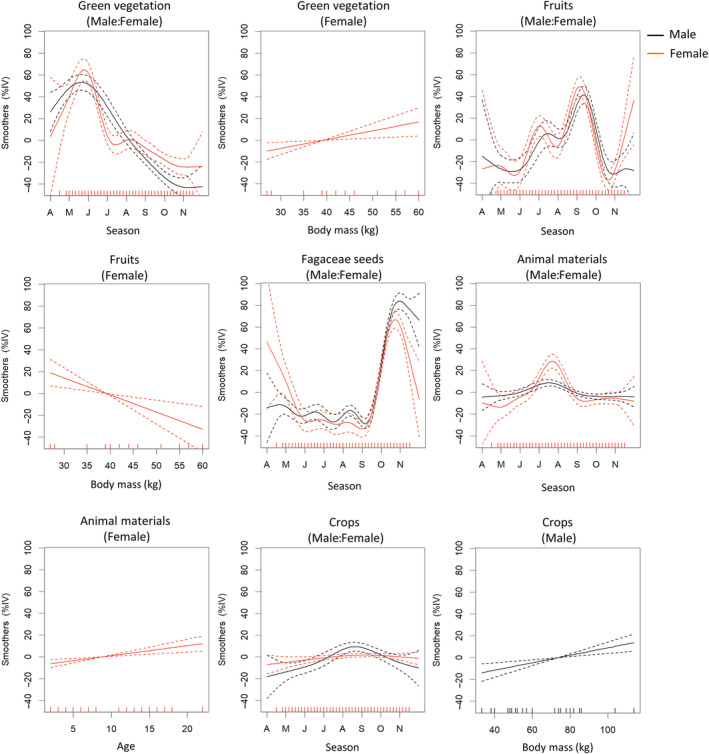
Seasonal trends in the percentage of important values (%IV) for each food category as a function of time (48‐time sections), body mass, and age of male and female Asiatic black bears (*Ursus thibetanus*) in Nagano Prefecture, Japan, based on the best‐fit models. The curves represent the estimated smooth function, and dotted lines indicate the 95% confidence intervals.

The AICc comparison of models with and without the random effect of individual ID and with and without temporal autocorrelation showed that the model including the random effect of individual ID and excluding temporal autocorrelation was optimal (Table 2). The GAMM analysis revealed striking variations in bear diets across seasons according to sex, age, and body mass classes (Table 2). However, despite these variations, most food categories exhibited broadly similar seasonal trends among these classes. Among males, the consumption of green vegetation gradually decreased from April to September and remained high in June and July. Conversely, females displayed a similar decreasing trend but exhibited a preference for fruits over green vegetation during June and July (Figure 2). Interestingly, large‐bodied females tended to prefer more green vegetation compared to fruits. The utilization of seeds from the Fagaceae family was similar between the sexes, with a substantial increase commencing in mid‐September. No discernable correlation emerged between diet, body mass, and age. Consumption of animal materials tended to increase from June and peaked in mid‐July for both sexes; however, overall consumption was higher in females than in males. Older females also exhibited a higher preference for animal material than younger females. Crops were consumed mainly from early August to late September by males, whereas no discernable effects of body mass or age were observed in females.

**TABLE 2:** Best‐fit generalized additive mixed models (GAMMs) displaying associations between %IV of each food category and season (smoother), body mass (smoother), and age (smoother) by sex in Asiatic black bears (*Ursus thibetanus*) in Nagano Prefecture, Japan, from 2016 to 2020.

Metrics	Best fit models	*R* ^2^	Df	AICc	Akaike weight
Male					
Green vegetation	sTime	0.537	6	1841.9	0.889
Fruits	sTime	0.417	6	1885.7	0.676
	sTime + sMass	0.434	8	1887.1	0.324
Fagaceae seeds	sTime	0.826	6	1685.1	0.863
Animal material	sTime	0.173	6	1476.6	0.875
Agricultural crops	sTime + sMass	0.140	8	1779.2	0.919
Female					
Green vegetation	sTime + sMass	0.598	8	1832.6	0.557
	sTime	0.589	6	1833.9	0.291
Fruits	sTime + sMass	0.480	8	1914.4	0.566
Fagaceae seeds	sTime	0.714	6	1848.7	0.650
	sTime + sMass	0.716	8	1850.7	0.242
Animal material	sTime + sAge	0.354	8	1704.1	0.752
Agricultural crops	Null	<0.01	4	1589.9	0.494

*Note*: Models are sorted by ascending AICc values and AIC weights. Only models with AICc values less than 2 are included to highlight the most candidate models. Adjusted *R* ^2^ values show the amount of variation explained in each model.

**FIGURE 3:**
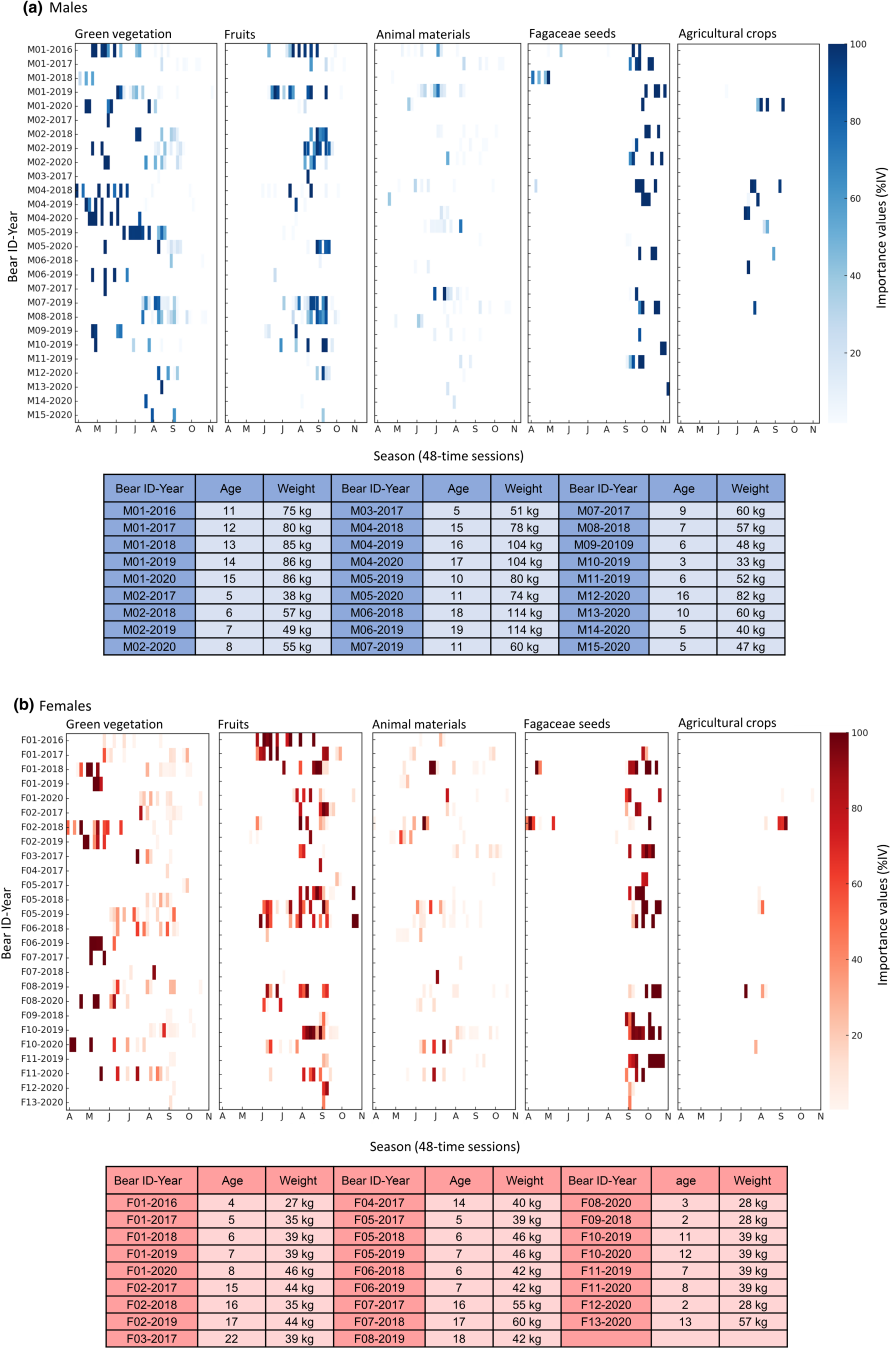
Seasonal dietary patterns of individually collared Asiatic black bears (*Ursus thibetanus*) in Nagano Prefecture, Japan, from 2016 to 2020 categorized by sex; (a) males and (b) females. The heatmaps represent the dietary composition (importance values%) across 48‐time intervals, with each interval corresponding to a specific month indicated on the horizontal axis (A, April; M, May; J, June; J, July; A, August; S, September; O, October; N, November). The vertical axis indicates the bears' ID and year.

Our GAMMs analysis explained (*R* ^2^) approximately half of the variance in %IV for each food category, except for animal materials and crops in males (Table 2). Additionally, there were notable differences that could not be attributed to sex, age, or body mass classes (Figure 3a,b). For example, specific male bears (M04 in 2018 and M05 in 2019) exhibited a strong reliance on green vegetation during early summer, whereas most other male bears consumed mixed diets comprising both green vegetation and fruit (e.g., M01 in 2016 and 2019). Among female bears, significant disparities in fruit consumption rates during late summer were evident among individuals, despite no significant differences in body mass or age (e.g., F01 in 2018 and F06 in 2018). Furthermore, we confirmed the presence of annual variations in the diets of the same individuals (F01 and M01).

## 4 DISCUSSION

Our study reanalyzes previously published data by Mori et al. (2019) to provide a more comprehensive understanding of the feeding habits of Asiatic black bears. By incorporating data from an additional 14 bears, resulting in a total sample size of 29 individuals, we aimed to investigate individual dietary differences and variations in feeding habits based on sex and body size. As anticipated, our analysis at the individual level revealed pronounced differences within and between these categories, which is consistent with the findings of previous studies, suggesting that individual bears adopt different foraging strategies depending on their sex, age, or body size (Lesmerises et al., 2015; Naganuma et al., 2020; Thiemann et al., 2011).

### 4.1 Population‐level diets

The primary reliance on green vegetation from April to mid‐June, continuing its importance until September, is due to the high nutritional value of young leaves and stems in spring and the efficient consumption of large quantities of mature leaves as the season progresses. During the spring, young leaves and stems offer a high crude protein content and low fiber levels, rendering them nutritionally valuable to bears (Furusaka et al., 2017). After early summer, although the fiber content in green vegetation increased, the leaves expanded in size, enabling bears to consume larger quantities in a single feeding (i.e., larger bite size). Previous studies report that grizzly bears (*U. a. horribilis*) tend to select food sources that are more efficient for foraging, requiring less search time and accommodating larger bite sizes (Welch et al., 1997). Thus, foraging for green vegetation after early summer retains its importance despite the diminished nutritional value.

The bimodal peak in fruit consumption during late June and early September indicates a strategic shift to high‐energy food sources when they become available. The initial peak coincided with fruits such as cherries and *Rubus* spp., whereas the subsequent peak correlated with dogwood, Japanese walnuts, and various other fruits. Given the increase in the number of ripening fruits from early August to mid‐September (Takahashi et al., 2008), it is not surprising that the second peak is larger than the first. Although most fleshy fruits contain a lower protein concentration, they offer a rich source of easily digestible carbohydrates that efficiently convert into fat (Dahle et al., 1998; Masaki et al., 2012). Additionally, Japanese walnuts, despite their hard and tight‐fitting shells, are rich in lipid (Chiba, 2016). Therefore, prior to the autumn season, fruits—especially Japanese walnuts and dogwood—comprised the most important food source for bears in the study area.

Between the two peaks in mid‐July and early August, bears in our study area scarcely foraged for fruits, instead shifting their dietary focus towards insects, particularly social insects. A previous study indicated that even if bears devoted a substantial portion of their active time to foraging for ants (approximately 180–300 kcal/day [7–8 h]), they could not obtain sufficient energy from these insects (Yamazaki et al., 2012). Furthermore, another study revealed that the energy balance of bears in the Ashio‐Nikko Mountains during the summer (especially in July and August) showed negative values and was the lowest during the active seasons when they extensively foraged for ants (Furusaka et al., 2019). Our findings align with the notion that summer presents significant challenges for bears in terms of food availability (Yamanaka et al., 2011).

Several studies have emphasized the importance accumulating fat reserves through foraging on hard mast species in preparation for hibernation (Clevenger et al., 1992; Hashimoto & Takatsuki, 1997; Nelson et al., 1983). Our data revealed a marked increase in the utilization of the seeds from the Fagaceae family, commencing in in mid‐September. This shift in dietary preference may be correlated with the ripening stage of oak species. Beginning in early September, the dry mass of oak on the branches increases rapidly, and the tannin content started to decline in mid‐September (Nakajima et al., 2012). However, the timing of bears consuming seed of the Fagaceae family in our study area did not coincide with the peak availability of dry oak mass or their lowest tannin levels. Because the availability of seeds that have fallen to the ground rapidly decreases due to competition with other animals (Shaw, 1968), it is likely that bears start feeding on seeds of the Fagaceae family before acorns fallen, oak dry mass has increased, and tannins have decreased to a certain extent.

### 4.2 Sex, age, and individual variations in diet

Recent nutritional studies have suggested that body size is one of the main factors contributing to differences in foraging strategies among individuals. For example, in the case of grizzly bears, small‐bodied individuals meet their nutrient requirements by consuming a higher proportion of small, sparsely distributed fruits owing to their lower total daily energy requirements (Welch et al., 1997). In contrast, large‐bodied bears cannot satisfy their nutrient requirements with fruit‐dominated diets owing to their high energy requirements. Therefore, they opt for energy‐rich alternatives such as salmon and ungulates (Costello et al., 2016; Robbins et al., 2007). Similarly, an earlier study on Asiatic black bears in the Ashio‐Nikko Mountains documented a greater use of sika deer by large adult male bears during early summer (Naganuma et al., 2020). Although our findings support the notion that body size plays a significant role in influencing dietary differences among individual bears, it is worth noting that energy‐rich foods (i.e., mammals) did not emerge as a distinguishing factor.

Our GAMM analysis revealed that male bears consumed more green vegetation during early summer (mid‐June to late July) compared to female bears. This period is marked by limited availability of energy‐rich fruits, necessitating that larger‐bodied males, with their higher energy requirements, opt for more readily available green vegetation despite its lower nutritional value compared to spring green vegetation. This strategy minimizes the energy expenditure associated with searching for widely scattered fruits, allowing them to meet their substantial energy needs more efficiently. Conversely, smaller‐bodied female bears consumed more fruits during the same period. Additionally, large‐bodied female bears tended to consume a greater quantity of green vegetation in contrast to their small‐bodied counterparts, leaned toward fruit consumption (Figure 2). This difference may be due to the lower total daily energy requirements of smaller‐bodied females, allowing them to rely on the scattered but energy‐rich fruits. Most Ursidae, including Asiatic black bears, exhibit sexual dimorphism (Christiansen, 2007), with male bears in our study area weighing approximately 1.56 times more than female bears (GLMM: *F*_1,57_ = 12.06, *p* = .01). Fruits available in early summer are limited to specific species such as cherry and *Rubus* spp. (Koike, 2010). Consequently, these fruits are not abundant enough to meet the high energy requirements of larger‐bodied males. This scarcity forces males and large‐bodied females to rely more on green vegetation, which, despite its lower energy density, is more consistently available during this period. However, why did male bears in our study area not exhibit a preference for mammals, such as sika deer, like those on Ashio‐Nikko Mountain? One possibility could be the disparity in the sika deer population density. Older, large‐bodied American black bears in Utah tend to exhibit more carnivorous tendencies than females in hunting units with high ungulate densities, but such trends are absent in hunting units with lower ungulate densities (Hatch et al., 2019). In the Ashio‐Nikko Mountains, a population‐level dietary study employing scat analysis demonstrated that sika deer constituted a significant portion of the bears' diet during the summer (Koike et al., 2016). In contrast, foraging of bears for mammals was rare in our study area, which features a low density of sika deer (T. Mori, personal observation). Thus, in regions with fewer predation opportunities, it is likely that there exists no discernable difference in mammalian foraging between sexes or based on body size. Additionally, as a substitute for high‐nutrient meat, males bears, particularly those with greater body mass, may turn to agricultural crops in late summer.

Female bears consumed more animal material, primarily insects, during early summer (July and August), consistent with reports from the Ashio‐Nikko Mountains, where stable isotopes of hair samples were investigated (Naganuma et al., 2020). Previous studies on American black bears and grizzly bears have also suggested that insects play a more significant role in the diets of small‐bodied black bears and female grizzly bears. Additionally, there appears to be an inverse relationship between insect consumption rates and body mass (Costello et al., 2016). The underlying reason for this phenomenon likely stems from the fact that large‐bodied bears are constrained by their food intake rate, especially when consuming ants (McLellan, 2011; Welch et al., 1997). This constraint could explain why small‐bodied bears, specifically female bears, demonstrated a higher inclination toward consuming ants compared to their male counterparts in our study area. However, the rationale behind older females tending to consume more animal material remains unknown.

Interestingly, the utilization pattern of seeds from the Fagaceae family exhibited uniformity among both male and female bears. In contrast, Mori et al. (2019) reported that female bears consumed Fagaceae family food sources earlier than male bears in a 2‐year study. It is well established that bear diets at the population level undergo annual fluctuations depending on fruit availability (e.g., Koike, 2010; Mattson et al., 1991). Since Mori et al. (2019) conducted their study over a relatively short period (2 years), certain environmental factors, such as food availability, may have prompted females to forage for Fagaceae earlier than males. Based on the average trends observed in relatively long‐term studies (5 years), no discernable differences between males and females emerged at the start of Fagaceae family food utilization.

Our GAMMs analysis explained half of the variance in %IV for each food category, indicating that factors beyond season, sex, age, and body mass contribute to individual dietary preferences. Individual diets are influenced by maternal learning, in addition to individual characteristics such as sex and age (Estes et al., 2003). Furthermore, female American black bears with cubs tend to consume more hares and *Vespidae* spp. to meet their nutritional requirements (Lesmerises et al., 2015). Therefore, we were unable to examine individual dietary differences from these perspectives. However, it is worth noting that two male bears (M04 and M05) exhibited a substantial reliance on green vegetation during early summer (Figure 3a). Typically, green vegetation‐dominated diets in the summer have only been reported in Asiatic black bears inhabiting the Sea of Japan side of Honshu and high‐altitude areas (Huygens et al., 2003; Mori et al., 2018). Our study provides evidence that intra‐individual variations in diet can surpass regional variations.

However, this does not imply that the remaining unexplained variability is solely attributable to individual differences. Several other factors could contribute to this unexplained variability, such as annual variations in food availability and intraspecific competition. Annual variations in food availability, such as the fluctuating abundance of fruit and mast, can significantly influence bear diets (Koike, 2010). These variations may affect some individuals more than others, depending on their foraging strategies and habitat use. Intraspecific competition can also play a role, with more dominant bears having better access to preferred food resources, while subordinate individuals may be forced to consume less preferred or more opportunistic foods (Levi et al., 2012).

In this study, we provide valuable insights into the individual‐level dietary preferences of bears. However, our study had certain limitations. First, although we used a GPS cluster‐based scat sampling method, the absence of fecal DNA analysis means we cannot definitively confirm that all scat samples were from the targeted collared bears. To improve accuracy in identifying individual animals, future studies should integrate fecal DNA analysis, enhancing the precision of data from GPS clusters and reducing the risk of misinterpretation. Second, our study used scat analysis to determine dietary patterns, which can be subject to biases (McLellan & Hovey, 1995). For instance, different food items vary in digestibility, leading to potential overrepresentation of harder‐to‐digest items like green vegetation and underrepresentation of meets (McLellan & Hovey, 1995). To mitigate these biases, future studies should consider complementary methods, such as direct observation (Tezuka et al., 2023) and stable isotope analysis. Third, the data on April and late October were sparse due to the difficulty in collecting samples during these periods. In April, many bears were still in hibernation, leading to fewer samples. Although this could affect the results, the diet in April and May is likely similar due to plant phenology, mitigating significant issues. Fourth, our study was limited by a small sample size and did not examine food availability, which prevented us from considering annual variations in diets and accounting for other factors contributing to individual dietary variation. Within our study area, we observed annual variations in the diets of individual bears (e.g., M01 and F01). These variations may account for the differences between the findings of this study and those reported by Mori et al. (2019). Furthermore, the degree of annual dietary variation may vary among individuals. For example, bears with diets predominantly consisting of green vegetation during the summer may not be as impacted by fluctuations in fruit availability. Consequently, they might exhibit relatively minor annual variations in their diets, especially until late summer, in contrast to other bears. Several other factors, such as intraspecific competition, an individual bear's experience, reproductive status, and maternal learning, can also influence individual differences. Therefore, future studies should consider the effects of annual variations, food availability, and other influences on the dietary composition of Asian black bears.

## AUTHOR CONTRIBUTIONS

**Tomoki Mori:** Conceptualization (lead); data curation (lead); formal analysis (lead); funding acquisition (supporting); investigation (lead); methodology (lead); project administration (lead); validation (equal); visualization (lead); writing – original draft (lead); writing – review and editing (lead). **Saki N. Mori:** Conceptualization (supporting); data curation (supporting); investigation (supporting); methodology (supporting); writing – original draft (supporting). **Shigeyuki Izumiyama:** Investigation (supporting); resources (lead); supervision (supporting); writing – review and editing (equal).

## CONFLICT OF INTEREST STATEMENT

The authors have no relevant financial or nonfinancial interests to disclose.

## Data Availability

The datasets generated and/or analyzed during the current study are available online in Dryad at https://datadryad.org/stash/share/Ngkb13P7tZgcqklu8EteZgYyGIZhQKOsqsi4G6VNVog.
